# IMPACT OF A TELEPHONE REMINISCENCE PROGRAM ON THE MENTAL HEALTH OF OLDER ADULTS: A RANDOMIZED WAIT-LIST CONTROL TRIAL

**DOI:** 10.1093/geroni/igae098.2855

**Published:** 2024-12-31

**Authors:** Juliette Shellman, Yuxuan Yang, Cameron Graham

**Affiliations:** University of Connecticut, Storrs, Connecticut, United States; University of Connecticut, Storrs, Connecticut, United States; University of Connecticut, Storrs, Connecticut, United States

## Abstract

There is increasing evidence that social isolation and loneliness have negative effects on the mental health of older adults. Reminiscence is one example of a cost-effective intervention shown to improve mental health and wellness. A double-blinded randomized controlled trial was employed to test the effectiveness of a telephone reminiscence intervention on mental health outcomes in community-dwelling older adults. The telephone-based program (STORII) is designed to provide a service that automatically calls older adults three times a week asking them meaningful questions about their lives. Responses are recorded over the phone and automatically transcribed. Participants in the intervention group received 3 phone calls per week for 6 weeks. The primary outcome, mental health, was measured by the Mental Health Continuum Scale (MHC). After baseline assessment, eligible participants (N=90) were randomized to either the telephone simple reminiscence intervention (n=41) or 6-week wait-list group (n=39). All participants in the wait-list control subsequently received the telephone reminiscence intervention. An ANCOVA model was run to measure the treatment effect of the intervention, controlling for differences in baseline demographic variables between the groups. Results showed a significant improvement in the psychological well-being dimension of the MHC. Findings support of the use of a telephone reminiscence program for improving the psychological well-being dimension of mental health in older adults. Non-significant findings in the social and emotional well-being dimensions may be due to the length of the intervention. No adverse reactions were reported by any of the participants. Clinical trial registry number NCT05977608.

